# Associated factors and persistence of palatal groove in preterm infants: a cohort study

**DOI:** 10.1186/s12887-016-0671-1

**Published:** 2016-08-24

**Authors:** Andréa A. O. Cortines, Luciane R. Costa

**Affiliations:** 1 University Hospital, Universidade Federal de Goias, Goiania, Goias Brazil; 2 Faculty of Dentistry, Universidade Federal de Goias, Primeira Avenida, s/n, Setor Universitario, Goiania, Goias 74605-220 Brazil

**Keywords:** Premature infant, Palate, Mouth abnormalities, Dentofacial deformities, Neonatal intensive care units, Intubation, Enteral nutrition

## Abstract

**Background:**

There is a lack of evidence on the relationship between prematurity and palatal abnormalities. The aim of this study was to evaluate the incidence of palatal groove, the associated factors and the persistence time in preterm infants from birth to 24 months of age.

**Methods:**

The children’s data, medical history and eating habits were collected using a questionnaire answered by the legal guardian and updated every dental visit. Natal and neonatal data were obtained from the medical records. During the orofacial examination, the presence or absence of a palatal groove was observed. In order to evaluate for associations between independent variables and the palatal groove, descriptive analyses and bivariate analyses were conducted using the Mann-Whitney, Pearson’s chi-squared and Fisher’s exact tests, when appropriate. The Poisson regression analysis was used to determine risk and protective factors for the occurrence of palatal groove. The significance level was 0.05. For the persistence of palatal groove, a survival analysis was used (Kaplan Meier method).

**Results:**

Seventy-four preterm infants were monitored. Palatal groove occurred in *n* = 19 (25.7 %) and persisted for an average time of 12 months. Bivariate analysis showed a significantly higher occurrence of palatal groove in girls (68.4 % vs 40 % with non-occurrence of palatal groove) as well as in infants that stayed longer in the neonatal intensive care unit (NICU) (median 37 days vs 20 days), that did not have exclusive breastfeeding (94.7 % vs 69.1 %), were intubated (median five days vs one day) or used an orogastric tube (median 33 days vs 15 days). The quantitative data for ‘NICU’, ‘intubation’ and ‘orogastric tube’ were correlated and estimated as risk factors for palatal groove formation in the unadjusted Poisson regression analysis.

**Conclusions:**

Palatal groove occur transiently in approximately one quarter of preterm infants, especially in infants that stay longer in the NICU, are intubated or use an orogastric tube.

## Background

Preterm birth of children is a global problem that afflicts approximately 5 to 18 % of the population [1]. One of the consequences of preterm birth (gestational age of less than 37 weeks or weighing less than 2,500 g) [1, 2] in the oral cavity is alteration of the palate [3]. It is assumed that anatomical changes to the palate may lead to increases in malocclusion such as cross bite, as well as abnormalities in the tooth eruption path, which impacts dental alignment; these problems can lead to a greater need for orthodontic treatment [4].

A secondary analysis of the *Etude Epidémiologique sur les Petits Ages Gestationnels* (EPIPAGE) preterm infant cohort study showed that, at five years of age, 3.7 % of children (*n* = 1,711) had some alteration to their palate morphology [3]. However, the type of palate alteration was not distinguished in that study [3], and they were visually identified by physicians at a later time after the perinatal events, which likely interfered with the results.

There is no accurate definition of a ‘normal’ palate in an infant, but a groove or sulci system printed on the alveolar ridge is the distinctive feature of the infant palate [5]. The literature has shown that factors such as gestational age, birth weight and intubation may be associated with changes in palatal morphology, such as asymmetry, posterior narrowing, increased anterior tilting, greater depth and the incidence of palatal groove [6–11]. This special kind of grooving, frequently associated with intubation [12, 13], can be described as a “narrow channel of variable depth located near the midline of the palate, extending from the incisive foramen to the soft palate” [14]. Allegedly, the palatal groove might have an impact on the width of the maxilla of children born preterm; this means that those children need a systematic follow-up during childhood to evaluate for potential malocclusions [12, 13].

Nevertheless, the aforementioned studies [6–11] are of a cross-sectional nature and show controversial results as to the risk factors and the duration of the changes. Therefore, the evidence regarding the impact of prematurity on palatal morphology is weak [4, 13]. Thus, this prospective cohort study monitored preterm infants from birth to observe the incidence of palatal groove, as well as associated factors and persistence time.

## Methods

This cohort study was approved by the Institutional Research Ethics Board (protocol #144/2011), and one of the parents of each child signed the informed and free consent form agreeing to participate in the study.

The monitoring of the children was performed in a university hospital in the mid-west region of Brazil, which has a Neonatal Intensive Care Unit (NICU) that receives newborns both internally and from other hospitals. The population attended to this hospital is generally of low socioeconomic level. After discharge from the NICU, the children are monitored on an outpatient basis by the medical team up to two years of age. The intervals between follow-up visits are established as needed by medical recommendations. The clinic operates twice a week.

For this study, all outpatient infants from that hospital service, born between February 2012 and December 2013 without facial deformities or congenital syndromes, were included, provided they were edentulous at their first dental care visit and their parents consented to participate. Children who could not be examined in the first dental care visit or did not attend any follow-up visits were excluded. The children were monitored between February 2012 and July 2014. The sample was non-probabilistic and consecutive. General data were obtained from birth, but the first dental assessment started when they began their follow-up consultations in the medical outpatient clinic.

Consultations with the dental team were held on the same day as consults scheduled with the medical team. At the first dental visit, the guardian filled out a questionnaire addressing the data, medical history and feeding habits of the child. At the follow-up visits, the medical, dental and nutrition histories were updated. Additionally, data about the gestational age and the infant’s condition at birth (weight, height, admission to NICU, ventilator support, feeding through orogastric tube) were obtained from the infant’s medical records. These data were obtained immediately after childbirth. Categorisation of the gestational ages was based on the criteria of the World Health Organization (WHO) [1]: Extremely preterm (< 28 weeks), very preterm (28 to < 32 weeks) and moderate to late preterm (32 to < 37 weeks). Birth weight was categorised as very low (< 1,500 g), low (1,500 to 2,499 g) and normal (≥ 2,500 g).

Afterwards, orofacial examination of the infant was performed. Three trained dentists alternated for the exam in pairs, so they reached a consensus on the diagnosis of palatal groove. The dentists were not blinded to the children’s medical history. Training of examiners to evaluate the persistence of the palatal groove consisted of a theoretical study on the diagnostic criteria based on slides with examples of different shaped grooves. Two out of the three examiners performed the oral assessment simultaneously. The dentist placed the child in a knee-to-knee position with the parent and examined its oral cavity with the aid of artificial light (headlamp) and a mouth mirror. When examining the palate, the presence or absence of a palatal groove was visually observed, using the description of Erenberg and Nowak [14]. The observations were recorded in a clinical chart prepared for this purpose.

The sample size was estimated based on a 25.0 % incidence of palatal groove in intubated preterm infants, compared with zero cases in non-intubated term infants [7]. Thus, for a cohort study with a significance level of 5 % and a test power of 80 %, it was determined that 68 infants are needed to identify associations between the incidence of palatal groove and intubation.

The data were analysed using IBM SPSS version 19. The quantitative variables showed non-normal distribution (except for birth weight). After descriptive analysis, a bivariate association between the independent variables and the incidence of palatal groove was sought, using the Mann-Whitney, Pearson’s chi-squared and Fisher’s exact tests, as appropriate. The correlations among quantitative variables were estimated with Spearman’s correlation test. The variables that were significantly associated with the occurrence of palatal groove in the bivariate analysis were included in the Poisson regression to identify risk and protective factors for the incidence of palatal groove in children born prematurely. The significance level was 0.05.

To evaluate the persistence of the palatal groove, survival analysis was used (Kaplan Meier method) in the software Prisma GraphPad. Based on the follow-up sessions, the time in months needed for disappearance of the palatal groove was recorded. The excluded cases were children who did not have the palatal groove as of the first consultation or who were not monitored long enough to observe the disappearance of this alteration.

## Results

A total of 177 children were evaluated for eligibility. Among them, 89 children were included. Of these children, fifteen were excluded due to the following criteria: five because it was not possible to complete the dental examination at the first visit and observe the presence or absence of the palatal groove, and ten because they did not come to follow-up visits. The results of this study thus refer to a cohort of 74 preterm infants.

The infants attended a median of six consultations (interquartile range = 4), with a median follow-up period of 11 months (interquartile range = 4). Other characteristics of the participants are described in Table 1.

**Table 1:** Participants. Participants’ characteristics (*n* = 74)

Preterm infants	*n* (%) or median (interquartile range)
Sex	
Male	39 (52.7 %)
Female	35 (47.3 %)
Age	
At birth (weeks)	32.0 (5.0)
In the first dental consultation (months)	3.0 (2.0)
In the last dental follow-up (months)	14.0 (14.0)
Birth weight (grams)	1542.5 (828.0)
Admitted to NICU	57 (77.0 %)
Were intubated	46 (62.2 %)
Use of orogastric tube	64 (86.5 %)
Breastfeeding	
Exclusive	18 (24.3 %)
Artificial	32 (43.2 %)
Mixed	24 (32.4 %)

At the first examination, the occurrence of a palatal groove was observed in 19 infants (25.7 %). In the bivariate analysis, the incidence of a palatal groove was significantly associated with the female sex, a longer stay in the NICU, intubation (higher frequency and duration), a longer use of an orogastric tube and the absence of exclusive breastfeeding (Table 2). Only one of the children with the palatal groove was exclusively breastfed, and the palatal groove persisted for 16 months in this girl and then disappeared; she was born with a very low birth weight, was very premature, and required admission to the NICU, orotracheal intubation and transitory orogastric feeding.

**Table 2:** Perinatal factors and palatal groove. Association of perinatal variables and occurrence of palatal groove in preterm infants (*n* = 74)

Variables	Palatal groove		*P*-value
	Yes *n* = 19	No *n* = 55	
Female sex, *n* (%)	13 (68.4 %)	22 (40.0 %)	0.038^a^
Gestational age, *n* (%)			0.354^b^
Extremely preterm (< 28 weeks)	5 (26.3 %)	7 (12.7 %)	
Very preterm (28 to < 32 weeks)	7 (36.8 %)	21 (38.2 %)	
Preterm (32 to < 37 weeks)	7 (36.8 %)	27 (49.1 %)	
Infant’s weight at birth, *n* (%)			0.094^b^
Very low (< 1500 grams)	13 (68.4 %)	22 (40.0 %)	
Low (1500 to 2499 grams)	4 (21.1 %)	25 (45.5 %)	
Normal (≥ 2500 grams)	2 (10.5 %)	8 (14.5 %)	
Infant’s age (months) at the first dental consultation, median (interquartile range)	3.0 (3.0)	3.0 (2.0)	0.286^c^
Admitted to NICU, *n* (%)	17 (89.5 %)	40 (72.7 %)	0.207^a^
For how long (days), median (interquartile range)^d^	37.0 (51.0)	20.0 (44.0)	0.005^c^
Neonate was intubated, *n* (%)	16 (84.2 %)	30 (54.5 %)	0.028^a^
For how long (days), median (interquartile range)^d^	5.0 (23.0)	1.0 (5.0)	0.005^c^
Use of orogastric tube, *n* (%)	19 (100.0 %)	45 (81.8 %)	0.056^a^
For how long (days), median (interquartile range)^d^	33.0 (40.0)	15.0 (36.0)	0.019^c^
Exclusive breastfeeding, *n* (%)	1 (5.3 %)	17 (30.9 %)	0.030^a^
Non-nutritive sucking, *n* (%)	13 (68.4 %)	33 (60.0 %)	0.591

^a^Chi-square, Fischer exact test

^b^Pearson chi-square

^c^Mann-Whitney

^d^Including those cases that were not exposed to the factors – NICU, intubation, orogastric tube

In the Poisson regression analysis, the odds ratio values were individually calculated for the variables that showed *P* < 0.05 in the bivariate analysis, considering the NICU, intubation and orogastric tube as the quantitative variables. More days spent in the NICU, intubated or using an orogastric tube were associated with an increased likelihood of developing a palatal groove (Table 3). As only the quantitative variables had *P* < 0.05 in the unadjusted analyses, we tested their correlation before proceeding with the adjusted regression analysis. The number of days in the NICU correlated with days intubated (Spearman’s rho = 0.75, *P* < 0.001) and days using an orogastric tube (rho = 0.88, *P* < 0.001). The number of days intubated also correlated with days using an orogastric tube (rho = 0.71, *P* < 0.001). So, an adjusted model was not estimated because of the significant correlations among these variables.

**Table 3:** Risk factors for palatal groove. Risk factors for the occurrence of palatal groove in preterm infants according to the Poisson regression

Variables	Unadjusted		
	OR^a^	95 % CI^b^	*P*-value
Female sex	2.29	0.86–6.11	0.097
Exclusive breastfeeding	0.18	0.02–1.35	0.095
Length of days			
Staying in NICU	1.014	1.003–1.024	0.012
Intubated	1.036	1.005–1.069	0.022
Using orogastric tube	1.018	1.002–1.034	0.025

^a^Odds ratio

^b^Confidence intervals at 95 %

**Fig. 1:**
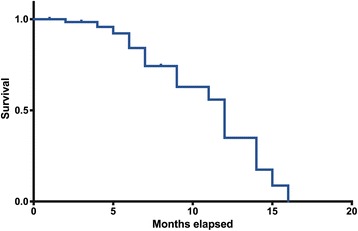
Incidence of palatal groove. Incidence of palatal groove according to the Kaplan-Meier curve

The palatal groove persisted for a median of 12 months (Fig. 1). There was no association between palatal groove persistence, sex, exclusive breastfeeding or admission to the NICU (*P* > 0.05). Disappearance of the groove was not observed in two of the 19 children. One was monitored until six months of age and the other until eight months of age.

## Discussion

The most significant finding of this longitudinal study was that palatal groove occurs transiently in approximately one quarter of preterm infants. The palatal groove especially affects infants that stay longer in the NICU, are intubated or use an orogastric tube. Thus, the clinical implication of this finding is that the palatal groove cannot be held responsible for morphological deficiencies of the palate that could result in malocclusions in deciduous, mixed or permanent dentition.

Surprisingly, in the bivariate analysis female children were more likely to have a palatal groove. In the EPIPAGE study, boys had more palatal alterations [3], but our results are not comparable because in the EPIPAGE study, the children were examined at five years of age by non-specialised physicians. It is also known that the jaw of boys is less mature than girls at 0.4 years of age [15]. The morphology of the normal palate in a newborn may differ according to sex and ethnicity [5]. Therefore, as the Brazilian population is characterised by a mixed ethnicity, this characteristic may have influenced this study’s results. However, further studies should seek further clarification regarding influence of sex and ethnicity.

Each additional day in the NICU increased the chance of a child having a palatal groove by 1 %. In fact, it is understood that admission to the NICU is an indicator of the severity of the preterm condition, as are intubation and use of an orogastric tube. Therefore, our results showed that intubation was associated with the occurrence of palatal groove: each additional day of intubation increased the risk of children having a palatal groove by 4 %. Intubation has been reported as a cause of oral deformities [7, 11], as has prolonged intubation [8, 11, 14]. Orotracheal intubation affects the growth of the palate, especially on the lateral process, leading to a smaller, narrower and deeper palate [16]. However, another study with 74 newborns with gestational ages from 25 to 41 weeks found that intubation for more than 10 days temporarily increases the depth of the palate by less than 2 mm, which is not equivalent to the palatal groove [9].

In this study, each additional day of orogastric tube use increased the risk of palatal groove development by 2 %. It is speculated that orotracheal and orogastric tubes can put pressure on the alveolar crest and the median region of the palate, negatively interfering with palatal growth and the “descent” of the jaw [10]. However, in one study, the use of an orogastric tube for up to 50 days was not associated with the occurrence of palatal groove in newborns and infants [14]. On the other hand, as the palatal groove can be observed in a slightly deep palate, it is assumed that it is not caused by direct pressure from the orotracheal tube [9]. A systematic literature review indicated that other possible causes for palatal groove include head flattening, tube pressure in the oral cavity and altered lingual function [13].

In this study, feeding was inversely associated with the occurrence of palatal groove in the bivariate analysis, but in the unadjusted regression it was not confirmed as a significant protection factor. Consequently, this association should be interpreted carefully. It is likely that more severely affected children did not breastfeed. So, along with a more consistent inclusion of the mode of feeding in future studies on palatal morphology of preterm infants [13], this result should also be verified in a study with a larger sample size.

Although infants with a median age of 14 months were monitored, our findings show that the palatal groove does not persist for long, although it was previously reported that palatal groove persist in children for two to 10 years of age [7, 8]. In addition, other changes in the palate associated with prematurity/intubation were reported in 11-year-old children [10]. However, our findings support the speculation that palate growth and remodelling can correct the occurrence of a palatal groove in preterm newborns associated with intubation [6]. A study involving 31 children two to five years old who were born preterm and were intubated detected that there was no palatal asymmetry [6]. In fact, jaw growth is quite fast in the first year of life and then decelerates in the following four years [15]. Another speculation is that, according to photographs of previously published cases [7], a deep palate could be mistaken for a palatal groove, leading to reports of palatal groove in older children. In this sense, it was agreed that there is no uniformity in the nomenclature of palate morphology and the definition of the palatal groove in infants [5, 12, 13].

One of the limitations of this study is that we did not have access to the oral conditions immediately after extubation and/or discharge, which could help in identifying the occurrence of an eventual palatal groove that may have disappeared by the first dental consultation. However, as the median age at the beginning of monitoring was three months, and most of the infants stayed in the NICU for a median time of approximately one month, it is believed that this factor did not negatively affect the results.

Another point to be discussed is the palatal groove diagnosis, which in this study was strictly visual, while other studies used models and other measuring devices [13]. However, the examiners in this study were systematically trained to identify the palatal groove. Moreover, due to the difficulties in thoroughly assessing the oral mouth of infants, as it can make them extremely irritated, we did not repeat oral exams to estimate inter- and intra-rater agreement. Instead, we opted for a consensus diagnosis between pairs of examiners, and therefore we believe this limitation does not invalidate our data.

Thus, the results of this study suggest that the occurrence of the palatal groove in infants is transient and associated with local traumatic factors, which most likely lead to growth of the oral soft tissues and “printing” of the tube on the palate. In any event, additional studies in infants are needed to investigate whether other changes in palate morphology involve environmental or genetic factors.

## Conclusions

Approximately one quarter of preterm infants had a palatal groove, which persisted for a median time of 12 months. The factors associated with the incidence of palatal groove were longer periods in NICU, intubation or use of orogastric tubes.

## Abbreviations

EPIPAGE: *Etude Epidémiologique sur les Petits Ages Gestationnels*
NICU: Neonatal Intensive Care Unit
WHO: World Health Organization
